# Correction: Compositional effect on the fabrication of Ag_*x*_Pd_1−*x*_ alloy nanoparticles on *c*-plane sapphire at distinctive stages of the solid-state-dewetting of bimetallic thin films

**DOI:** 10.1039/c8ra90014k

**Published:** 2018-02-15

**Authors:** Puran Pandey, Sundar Kunwar, Mao Sui, Sushil Bastola, Jihoon Lee

**Affiliations:** College of Electronics and Information, Kwangwoon University Nowon-gu Seoul 01897 South Korea jihoonleenano@gmail.com; Institute of Nanoscale Science and Engineering, University of Arkansas Fayetteville AR 72701 USA

## Abstract

Correction for ‘Compositional effect on the fabrication of Ag_*x*_Pd_1−*x*_ alloy nanoparticles on *c*-plane sapphire at distinctive stages of the solid-state-dewetting of bimetallic thin films’ by Puran Pandey *et al.*, *RSC Adv.*, 2017, **7**, 55471–55481.

Errors were present in the published article and ESI. The errors in the article are in the plots of SAR and coverage in Fig. 6(m) and (n) and the corrected figure is shown below. At the same time, Fig. 6(l) has been edited in order be consistent with Fig. 6(m) and (n). Specifically, the blue lines denote “Pd_0.25_Ag_0.75_” and the black lines “Pd_0.75_Ag_0.25_”. In the ESI summarized values of *R*_q_ in Table S7 were incorrect and the ESI document is now replaced.

**Fig. 6:**
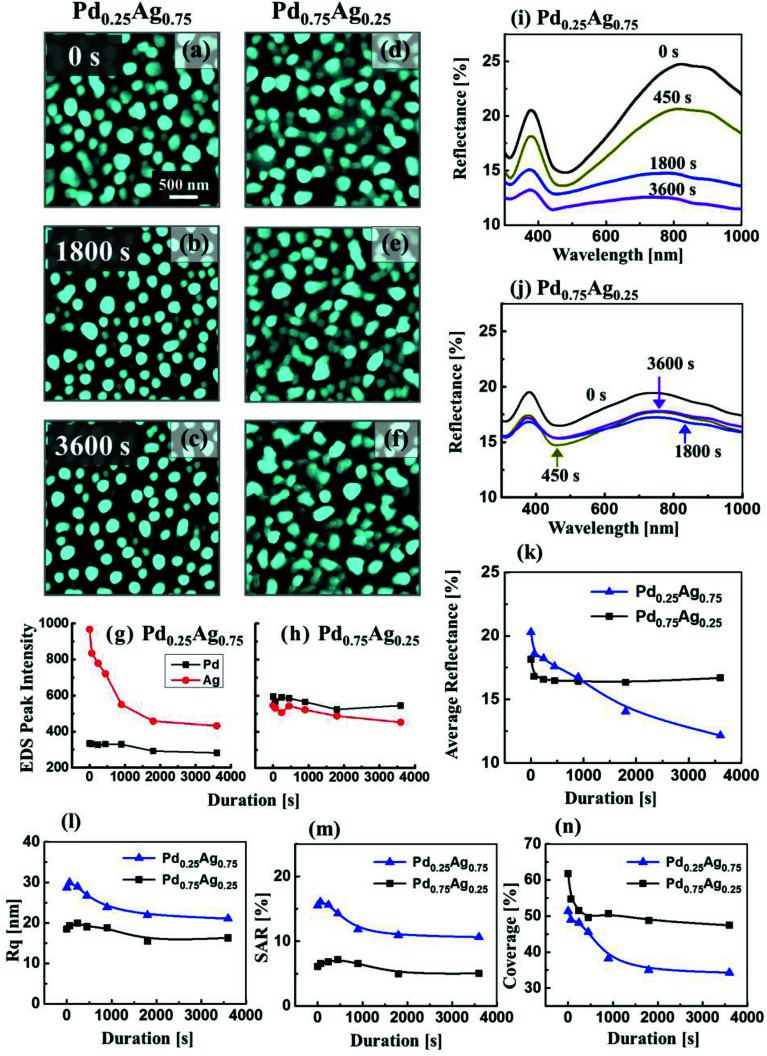
Evolution of Ag–Pd bimetallic nanostructures by the variation of annealing durations between 0 and 3600 s at 650 °C with the deposition thickness of 10 nm and distinct bilayer composition as labelled. (a)–(f) AFM top-views of 3 × 3 μm^2^. (g) and (h) Summary of EDS intensities of Ag Lα1 and Pd Lα1 with respect to the annealing durations. (i) and (j) Reflectance spectra of Ag–Pd nanostructures. (k) Summary plot of average reflectance. (l)–(n) Summary plot of *R*_q_, SAR and coverage plots with respect to the annealing duration.

The Royal Society of Chemistry apologises for these errors and any consequent inconvenience to authors and readers.

## Supplementary Material

